# Trends in willingness to receive COVID-19 vaccines among healthcare workers in India: Findings from repeated cross-sectional national surveys

**DOI:** 10.3389/fpubh.2022.994206

**Published:** 2022-10-03

**Authors:** Bijaya Kumar Padhi, Venkatesan Chakrapani, Madhu Gupta, Nikita Sharma, Binod Kumar Patro, Sitanshu Sekhar Kar, Ritesh Singh, Star Pala, Lalit Sankhe, Bhavesh Modi, Surya Bali, Neeti Rustagi, Lovely Jain, Jatina Vij, Prakasini Satapathy, Kapil Goel, Vineeth Rajagopal, Tanvi Kiran, Arun Kumar Aggarwal

**Affiliations:** ^1^Department of Community Medicine and School of Public Health, Post Graduate Institute of Medical Education and Research, Chandigarh, India; ^2^Centre for Sexuality and Health Research and Policy, Chennai, India; ^3^Department of Community and Family Medicine, All India Institute of Medical Sciences, Bilaspur, India; ^4^Department of Community and Family Medicine, All India Institute of Medical Sciences, Bhubaneswar, India; ^5^Department of Preventive and Social Medicine, Jawaharlal Institute of Postgraduate Medical Education and Research, Puducherry, India; ^6^Department of Community and Family Medicine, All India Institute of Medical Sciences, Kalyani, India; ^7^Department of Community Medicine, North Eastern Indira Gandhi Regional Institute of Health and Medical Sciences, Shillong, India; ^8^Department of Community Medicine, Grant Medical College, Mumbai, India; ^9^Department of Community and Family Medicine, All India Institute of Medical Sciences, Rajkot, India; ^10^Department of Community and Family Medicine, All India Institute of Medical Sciences, Bhopal, India; ^11^Department of Community Medicine and Family Medicine, All India Institute of Medical Sciences, Jodhpur, India; ^12^Regional Virus Research and Diagnostic Lab, Department of Virology, Post Graduate Institute of Medical Education and Research, Chandigarh, India

**Keywords:** vaccine acceptance, intention, hesitancy, domestic vaccine, trust

## Abstract

**Background:**

COVID-19 vaccination of the healthcare workers (HCWs) is a key priority in the fight against the SARS-CoV-2 pandemic. India launched its COVID-19 vaccination program in January 2021. We aimed to understand the trends in willingness to receive COVID-19 vaccines and its associated factors among HCWs in India.

**Methods:**

Using a repeated cross-sectional survey design, we collected information from HCWs in three critical time points: before (*n* = 937, October 2020), during (*n* = 1346, January 2021); and after (*n* = 812, May 2021) the introduction of COVID-19 vaccines in India. The third survey coincided with the peak of the second wave of COVID-19 pandemic in India.

**Findings:**

Of the study participants, 43.7, 60.2, and 73.2% were willing to receive COVID-19 vaccines during the first, second and third rounds of surveys, respectively. In multivariable logistic regression analysis, participants who trusted the health care system were more likely to report willingness to receive a COVID-19 vaccine; medical trust emerged as a significant factor in all the three rounds of surveys (First survey—aOR: 2.24, 95% CI: 1.67–2.99; Second survey—aOR: 3.38, 95% CI: 2.64-4.33; Third survey—aOR: 2.54, 95% CI: 1.65–3.91). Having confidence in domestic vaccines (Second survey—aOR: 2.21, 95% CI: 1.61–3.02; Third survey—aOR: 2.05, 95% CI: 1.24–3.37); and high perceived risk of contracting COVID-19 (Second survey—aOR: 1.48, 95% CI: 1.13–1.93; Third survey—aOR: 2.02, 95% CI: 1.31–3.13) were found to be associated with willingness to receive vaccines. Among socio-demographic characteristics, being married (aOR: 1.71, 95% CI: 1.08–2.71) and having high socio-economic status (aOR: 3.01, 95% CI: 1.65–5.51) emerged as significant factors associated with willingness to receive COVID-19 vaccines in the third round of the surveys.

**Interpretation:**

Willingness to receive COVID-19 vaccine increased with time, as the severity of the pandemic increased. To increase COVID-19 acceptance and coverage among HCWs, it is important to instill confidence in domestic vaccines and assist in accurate assessment of risk toward contracting COVID-19 infection.

## Introduction

Globally, severe acute respiratory syndrome (SARS-CoV-2) has affected 170 million people and caused 3.5 million deaths (1). Almost all the countries have suffered a major blow to their health systems and economies. India is among the top three countries reporting the highest number of COVID-19 cases in the world, with 247,968,227 confirmed cases including 5,020,204 deaths till November 4, 2021 (2). Under such gloomy circumstances, the COVID-19 vaccines have emerged as a ray of hope for halting the pandemic (3, 4). As the scientific world raced toward developing a vaccine, many vaccine candidates appeared on the horizon. The clinical trials of vaccine candidates from Pfizer/BioNtech, Moderna, Oxford/AstraZeneca, and Johnson & Johnson's Janssen Pharmaceuticals demonstrated their immunogenicity, safety, and efficacy (5–8). As the pandemic worsened, some vaccines were authorized for emergency use (9–12). India launched its COVID-19 vaccination drive with two vaccines, namely CoviShield (Serum Institute of India) and Covaxin (Bharat Biotech), on January 16, 2021 (13). In the first phase, the healthcare workers (HCWs) and frontline workers were vaccinated. Although HCWs can serve as ambassadors of COVID-19 vaccine acceptance, surveys have found low acceptance levels among HCWs; and many people remain skeptical about the safety and efficacy (14, 15).

The availability of vaccines does not guarantee uptake. In the 2009 influenza-A pandemic, less than one-fourth of the Americans were vaccinated during the first year (16). Low uptake of influenza vaccine was reported among HCWs (25%) in China (17). During the H1N1 Influenza pandemic, the intention of people to get vaccinated mirrored the severity of the pandemic and its decline, ultimately leading to a reduction in vaccine intent from 50 to 16% within 10 months (18). Despite the USA reporting the highest number of confirmed cases of COVID-19 in 2020, only half of the medical students expressed willingness to participate in a vaccine trial and 23% were unwilling to get vaccinated (19). After the first wave of the pandemic in India, vaccine hesitancy was 10.6% among medical students (20). The success of the COVID-19 vaccination program will depend on the HCWs' intention to receive vaccines.

Globally, vaccination rates have stagnated for the past few years (21). Recently, outbreaks of vaccine-preventable diseases such as measles and mumps in the USA were attributed to vaccine hesitancy and refusal (22). The World Health Organization has declared vaccine hesitancy among one of the top 10 threats to global health (23). The COVID-19 vaccine intent may also decline in the post-pandemic period due to a reduction in stress related to working conditions (24). Although few cross-sectional studies have reported the intention of COVID-19 vaccines among HCWs in India during the first wave, until now there is no information on the effect of the second wave of COVID-19 pandemic on vaccine acceptance. Moreover, the determinants of vaccine hesitancy may change with time as the COVID-19 pandemic advances. Accordingly, in this paper we report the trends in willingness to receive COVID-19 vaccine and its associated factors among healthcare workers during the first and second waves of COVID-19 pandemic in India.

## Methods

### Study design and participants

Three repeated cross-sectional surveys were conducted on the acceptability of COVID-19 vaccines among HCWs in India. The first survey was conducted in October 2020; the second survey in January 2021, and the third in May 2021. The first survey period corresponded with the period of the first wave of pandemic, before the launch of COVID-19 vaccination drive in India. The second survey period corresponded with the end of the first wave and the launch of COVID-19 vaccines in India. The third round of survey corresponded with the peak of the second wave of COVID-19 pandemic (25). We recruited 937, 1346 and 812 participants for the first, second and third rounds of surveys, respectively. As there was no prior data available at the time of design of the study on COVID-19 vaccine acceptance among healthcare workers, we relied on the standard parametric approach for a choice probability to approximate the minimum sample size. To be conservative, we calculated the minimum sample size (*N* = 768) on the assumption that COVID-19 vaccine acceptance level will be 50% (*p* = 50%); with absolute precision of 5 and 95% confidence interval. We considered a design effect of 2.0 given the non-random nature of suervey sampling. The OpenEpi 3.01 was used for calculating the sample size. The survey instruments were developed by a literature review of similar studies, and has been described elsewhere (26, 27). Study participants were recruited through convenience and snowball sampling. Study participants were invited through email and social media such as Twitter, Facebook, and the WhatsApp. Key healthcare forums were also targeted to recruit study participants. Efforts were made to recruit participants across major geographic regions including northern, southern, eastern, western, central and north-eastern India. The survey was administered in English language.

## Measures

### Outcomes

The key outcome measure was participant's intention or willingness to receive COVID-19 vaccines. The responses were recorded on a three-point Likert scale (accept, refuse, and undecided).

### Co-variables

We collected sociodemographic characteristics such as age, gender, marital status, education level, place of residency, occupation sector (government vs. private), and social status. Information about history of exposures to COVID-19; perceived risk of infection; trust in the healthcare system; and perception on domestic vaccines were captured.

### Statistical analyses

Descriptive statistical analysis was performed by cross-tabulating demographic characteristics with the primary outcome variable. Chi-square tests were performed for bivariate analysis (cross-tabulation) between the outcome variable (willingness to vaccinate) with all potential explanatory variables. Willingness to receive COVID-19 vaccines was measured as participants who reported to accept the vaccine at the time of survey. We performed multivariable logistic regression analysis to compute adjusted odds ratio (aOR) and 95% confidence interval (CI). We included only significant variables from the bivariate analysis in the multivariable model. An association was considered to be statistically significant if the two-tailed *p*-values was < 0.05. Stata 15.0 software (StataCorp LP, Texas, USA) was used for all statistical analyses.

### Ethical considerations

The study protocol was reviewed and approved by the Institutional Research Ethics Committee of Post-Graduate Institute of Medical Education & Research (PGIMER), Chandigarh, India, and electronic informed consent was obtained from all participants. De-identified data were used for analysis, interpretation, and reporting.

## Results

Between October 2020 and May 2021, a total of 3,095 HCWs responded to the three surveys. The sociodemographic characteristics of the participants showed that about half of them were aged 18–29 years in the first (43.0%), second (47.2%) and third surveys (53.0%), with a relatively higher proportion of females (59.6, 60.4, and 53.0%), married persons (55.6, 53.3, and 60.1%), and those with a postgraduate medical degree (40.4, 42.2, and 61.9%). Most participants (70.1, 75.2, and 56.6%) belonged to middle socio-economic status with 45.5, 47.3, and 43.8% reporting a family income of more than 50,000 rupees in the three rounds of the survey. Of the 937, 1,346 and 812 HCWs, 40.4, 33.1, and 45.0% worked in government sector, and 53.9, 48.0, and 41.2% were from rural areas in the first, second and third rounds of surveys, respectively (Table 1).

**Table 1:** Socio-demographic characteristics of the study participants.

**Variables**	**First survey (*n =* 937)**	**Second survey (*n =* 1,346)**	**Third survey (*n =* 812)**
Age (in years)			
18–29	403 (43.01%)	636 (47.25%)	431 (53.08%)
30–49	362 (38.63%)	634 (47.10%)	361 (44.46%)
Above 50	172 (18.36%)	76 (5.65%)	20 (2.46%)
Gender			
Man	378 (40.34%)	532 (39.52%)	381 (46.92%)
Woman	559 (59.66%)	814 (60.48%)	431 (53.08%)
Highest level of education			
Primary school or below	49 (5.23%)	25 (1.86%)	75 (9.24%)
High school/diploma	361 (38.53%)	303 (22.51%)	41 (5.05%)
Undergraduate	148 (15.80%)	449 (33.36%)	190 (23.40%)
Postgraduate	379 (40.45%)	569 (42.27%)	503 (61.95%)
Marital status			
Single	521 (55.60%)	718 (53.34%)	488 (60.10%)
Married	416 (44.40%)	628 (46.66%)	324 (39.90%)
Family size			
Five and below	689 (73.53%)	1,048 (77.86%)	683 (84.11%)
Six and above	248 (26.47%)	298 (22.14%)	129 (15.89%)
Place of work			
Government sector	379 (40.45%)	858 (65.15%)	366 (45.07%)
Private sector	558 (59.55%)	459 (34.85%)	204 (25.12%)
Family income (INR)			
Below 10,000	146 (15.58%)	168 (12.48%)	185 (22.78%)
11,000–20,000	118 (12.59%)	238 (17.68%)	105 (12.93%)
21,000–50,000	246 (26.25%)	303 (22.51%)	166 (20.44%)
Above 50,000	427 (45.57%)	637 (47.33%)	356 (43.84%)
Social status			
Low	99 (10.57%)	143 (10.62%)	194 (23.89%)
Middle	657 (70.12%)	1,013 (75.26%)	460 (56.65%)
High	181 (19.32%)	190 (14.12%)	158 (19.46%)
Geographical regions			
Eastern	230 (24.55%)	278 (20.65%)	154 (18.97%)
Western	79 (8.43%)	108 (8.02%)	130 (16.01%)
Northern	356 (37.99%)	561 (41.68%)	217 (26.72%)
Southern	112 (11.95%)	204 (15.16%)	205 (25.25%)
Central	88 (9.39%)	103 (7.65%)	59 (7.27%)
North-east	72 (7.68%)	92 (6.84%)	47 (5.79%)
Area of residence			
Urban	432 (46.10%)	699 (51.93%)	477 (58.74%)
Rural	505 (53.90%)	647 (48.07%)	335 (41.26%)

Table 2 presents the changes in willingness to accept COVID-19 vaccines, contact history with COVID-19 patients, risk perception, and vaccine preferences. We observed a steady increase in the intention to receive COVID-19 vaccines during the period of three surveys (43.7, 61.1, and 73.2%), with a 30% increase in willingness to receive vaccines between the first and third surveys. Between the first and third surveys, there was a significant increase in risk perception (67.5 vs. 81.1%), exposure to a confirmed COVID-19 patient (29.9 vs. 55.4%), and increased knowledge of COVID-19 (93.3 vs. 99.1%) and development of vaccines (86.7 vs. 92.6%). However, trust in the healthcare system (62.9 vs. 30.7%) was found to be lower between the first and third surveys (*p* < 0.001). Confidence in domestic vaccines was found to be unchanged (23.8, 25.3, and 25.8%) in the three surveys.

**Table 2:** Change over time in willingness to accept COVID-19 vaccine among healthcare worker during first and second wave of COVID-19 pandemic.

**Variables**	**First survey (*n =* 937)**	**Second survey (*n =* 1346)**	**Third survey (*n =* 812)**	***P*-value**
Willingness to vaccinate				< 0.001
Accept	410 (43.76%)	810 (60.18%)	595 (73.28%)	
Refuse	213 (22.73%)	154 (11.44%)	150 (18.47%)	
Undecided	314 (33.51%)	382 (28.38%)	67 (8.25%)	
Exposed to COVID-19 cases*				< 0.001
No	656 (70.01%)	888 (65.97%)	362 (44.58%)	
Yes	281 (29.99%)	458 (34.03%)	450 (55.42%)	
Knowledge about COVID-19				< 0.001
No/not sure	62 (6.62%)	77 (5.72%)	7 (0.86%)	
Yes	875 (93.38%)	1,269 (94.28%)	805 (99.14%)	
Knowledge on development of COVID-19 vaccines			
No/not sure	124 (13.23%)	161 (11.96%)	60 (7.39%)	
Yes	813 (86.77%)	1,185 (88.04%)	752 (92.61%)	
History of vaccine hesitancy				< 0.001
Yes	135 (14.41%)	219 (16.27%)	279 (34.36%)	
No	802 (85.59%)	1,127 (83.73%)	533 (65.64%)	
Perceived risk of COVID-19 infection				< 0.001
Yes	633 (67.56%)	955 (70.95%)	659 (81.16%)	
No	304 (32.44%)	391 (29.05%)	153 (18.84%)	
Trust in the healthcare system				< 0.001
No	347 (37.03%)	485 (36.03%)	562 (69.21%)	
Yes	590 (62.97%)	861 (63.97%)	250 (30.79%)	
Confidence in domestic vaccines				< 0.001
Better	223 (23.80%)	341 (25.33%)	210 (25.86%)	
Neutral	338 (36.07%)	450 (33.43%)	423 (52.09%)	
Worst	376 (40.13%)	555 (41.23%)	179 (22.04%)	

P-values by Chi^2^ test for binary/categorical variables.

*Exposed to the COVID-19 cases: HCW who came in contact with a confirmed COVID-19 cases during treatment, travel and or residence.

Table 3 shows the findings from multivariable logistic regression analysis of the factors influencing COVID-19 vaccine uptake among HCWs. Trust in the healthcare system was consistently associated with the intention to receive COVID-19 vaccines [(aOR: 2.24, 95% CI: 1.67–2.99); (aOR: 3.38, 95% CI: 2.64–4.33), and (aOR: 2.54, 95% CI: 1.65–3.91)] in all the three surveys. Participants with a higher risk perception were likely to be vaccinated in the second (aOR: 1.48, 95% CI: 1.13–1.93) and third surveys (aOR: 2.02, 95% CI: 1.31–3.13). Confidence in domestic vaccines was found to be a significant factor associated with the acceptance of COVID-19 vaccines. Those participants who were married (aOR: 1.71, 95% CI: 1.08–2.71) and who belong to high socio-economic status (aOR: 3.01, 95% CI: 1.65–5.51) were also more likely to report willingness to receive COVID-19 vaccine in all the three surveys. When compared to those aged 49 and below, participants aged 50 and above were found to have lower odds of accepting the vaccine (aOR: 0.21, 95% CI: 0.07–0.61; *p* = 0.004) in the third round of survey.

**Table 3:** Factors influencing COVID-19 vaccine uptake among HCWs: multivariable logistic regression analysis^+^.

	**Willingness to receive COVID-19 vaccines**					
	**First survey (*N =* 937)**		**Second survey (*N =* 1,346)**		**Third survey (*N =*812)**	
	**aOR (95% CI)**	***p*-value**	**aOR (95% CI)**	***p*-value**	**aOR (95% CI)**	***p*-value**
Trust in healthcare system						
No	Ref		Ref		Ref	
Yes	2.24 (1.67, 2.99)	0.000	3.38 (2.64, 4.33)	0.000	2.54 (1.65, 3.91)	< 0.001
Exposed to COVID-19 cases						
No	Ref		Ref		Ref	
Yes	0.99 (0.73, 1.34)	0.972	0.78 (0.60, 1.00)	0.053	0.75 (0.52, 1.07)	0.120
History of vaccine hesitancy						
Yes	Ref		Ref		Ref	
No	1.13 (0.76, 1.67)	0.263	0.83 (0.59, 1.15)	0.263	0.78 (0.54, 1.13)	0.196
Confidence in domestic vaccines						
Worst	Ref		Ref		Ref	
Neutral	0.99 (0.69, 1.43)	0.994	1.94 (1.47, 2.55)	0.000	1.62 (1.05, 2.49)	0.028
Better	1.30 (0.95, 1.78)	0.100	2.21 (1.61, 3.02)	0.000	2.05 (1.24, 3.37)	0.005
Perceived risk of COVID-19 infection						
No	Ref		Ref		Ref	
Yes	1.27 (0.942 1.716)	0.100	1.48 (1.13, 1.93)	0.004	2.02 (1.31, 3.13)	0.001
Age (in years)						
18-29	Ref		Ref		Ref	
30-49	0.73 (0.48, 1.11)	0.151	1.21 (0.82,1.78)	0.325	1.57 (0.98, 2.50)	0.058
Above 50	0.41 (0.26, 0.64)	0.000	1.35 (0.72, 2.52)	0.340	0.21 (0.07, 0.61)	0.004
Gender						
Man	Ref		Ref		Ref	
Woman	1.40 (1.04, 1.88)	0.023	1.07 (0.83, 1.37)	0.589	1.46 (0.99, 2.16)	0.054
Marital status						
Single	Ref		Ref		Ref	
Married	1.05 (0.71, 1.56)	0.788	1.05 (0.72, 1.53)	0.772	1.71 (1.08, 2.71)	0.020
Place of residence						
Urban	Ref		Ref		Ref	
Rural	0.88 (0.67, 1.16)	0.373	1.12 (0.88, 1.42)	0.326	1.13 (0.80, 1.58)	0.474
Highest level of education						
Primary school	Ref		Ref		Ref	
Diploma/High School	0.87 (0.45, 1.68)	0.694	0.86 (0.35, 2.14)	0.760	1.29 (0.66, 2.52)	0.448
Undergraduate	1.01 (0.50, 2.04)	0.962	1.53 (0.60, 3.84)	0.365	2.16 (0.86, 5.41)	0.098
Postgraduate	0.81 (0.42, 1.55)	0.530	1.59 (0.63, 4.00)	0.317	1.18 (0.77, 1.81)	0.436
Social status*						
Low	Ref		Ref		Ref	
Middle	0.68 (0.44, 1.04)	0.075	0.68 (0.44, 1.04)	0.075	1.33 (0.85, 2.09)	0.206
High	1.13 (0.66, 1.92)	0.640	1.13 (0.66, 1.92)	0.640	3.01 (1.65, 5.51)	< 0.001

*Self-reported information about perceived social status in participants neighborhoods.

^+^We included only significant variables from the bivariate analysis in the multivariable model.

## Discussion

This is probably the first study from India that documented the trends in COVID-19 vaccine acceptance among HCWs and identified significant correlates of acceptance at three critical time points of the COVID-19 pandemic in India. A repeated cross-sectional survey design has advantages over an one-time cross-sectional survey, especially while investigating changes in the health-related behavioral outcomes. Our study reported an increment of 30% (43.7 vs. 73.3%, *p* < 0.001), in the first and third rounds) of acceptance of COVID-19 vaccines before and after the introduction of vaccines in India, which may be as a results of the massive campaigns on the COVID-19 vaccinations after the introduction of the vaccines might influence participants' willingness. There was a significant improvement in the knowledge about COVID-19 and the development of COVID-19 vaccines between the first and third surveys. Trust in the healthcare system, trust in domestic vaccines, and high-risk perception emerged as key predictors of COVID-19 vaccine acceptance among HCWs in India. The other significant predictors during the third round of the survey were higher social status (aOR: 3.01; 95% CI: 1.65–5.52) and being married (aOR: 1.72; 95% CI: 1.09–2.71).

During the first wave of the pandemic, a high rate of intention to receive vaccines was observed in China (83.5%), Malaysia (83.3%), and the USA (78%) (28–30). The vaccine acceptance rates among HCWs varied between 27.7 and 78.1% for COVID-19 vaccines (31). In the present study, after the first wave of COVID-19 pandemic (2020), about 60.2% reported willingness to receive COVID-19 vaccines while 73.3% were willing during the second wave in 2021. In China, the vaccine acceptance rate was 91.3% in March, 83.5% in May, and 88.6% in June, 2020 (28, 32). Studies reported the vaccine acceptance rate of 79% in April, 83% in May, 64% in July, and 71.7% in the fall of 2020 in the United Kingdom (31). In the present study, the exposure of HCWs to COVID-19 cases significantly increased from 34.3% in the first wave compared to 55.1% in the second wave. This increase in exposure of cases probably contributed to increase in risk perception and in turn to increase in acceptance of COVID-19 vaccines. This hypothesis is supported by findings from other studies that reported that people who perceived themselves at high risk of COVID-19 infection are more likely to get vaccinated (20, 27, 32). In the present study, we found that those HCWs who perceived themselves at high risk had higher odds of COVID-19 vaccine acceptance than those who had lower risk perception (aOR: 1.48 and 95% CI: 1.14–1.93, during the second round, and aOR: 2.03 and 95% CI: 1.31–3.14, during the third round).

During the first round, age, gender, education, marital status, area of residence, socioeconomic status had no influence on COVID-19 vaccine uptake among healthcare workers. In the second round, participants older than 50 years had lower odds of COVID-19 vaccine acceptance when compared to younger participants (aOR: 0.22, 95% CI: 0.08-0.62). It is not clear what could have contributed to this difference. Similar findings were reported in a Malaysian study where vaccine hesitancy among people aged 60 years and above was five times than the young people aged 18–29 years (29). In the present study, participants with higher socioeconomic status were three times more likely to be willing to receive COVID-19 vaccines (aOR: 3.02, 95% CI: 1.65–5.52) than those with lower socioeconomic status, and married participants were 1.7 times more likely to accept COVID-19 vaccines than those who were single. Similar findings were also reported in China and Saudi Arabia that marital status can influence the intention to accept the vaccine (27, 32). It is possible that married people feel that they have a responsibility to protect their family members and more likely to accept COVID-19 vaccines.

Studies have shown that trust in the healthcare system increases the likelihood of acceptability of COVID-19 vaccines (27, 33). In the present study, the trust of healthcare workers in the healthcare system declined (63.9% in the first survey to 30.8% in the third survey, *p* < 0.001). The second wave of the SARS-CoV-2 pandemic strained the healthcare system in India, leading to a shortage of life-saving drugs, medical supplies, oxygen and ultimately resulted in greater mortality than the first wave. This might have contributed to the decrease in trust on the healthcare system. Also, since HCWs were among the first set of people to receive vaccines in India, it is possible that they were concerned about the safety of vaccines, a concern reported in studies from other countries (34). During the first wave of the pandemic, only one-fourth of the HCWs trusted the domestic vaccines while 34.5% were neutral about them. This is in contrast to a study from China that reported 94.8% of the participants had confidence on domestic vaccines (28). After the announcement about COVID-19 vaccines for healthcare workers, the mistrust in the domestic vaccines reduced from 41.2% in the second round to 22.0% in the third round (during the second wave of COVID-19 pandemic). In the present study, HCWs who had confidence on domestic vaccines were two times more likely to get vaccinated than those who did not have confidence on domestic vaccines.

Numerous studies highlighted major barriers in accepting COVID-19 vaccines which include side-effects of vaccine, speed of development of the vaccine, uncertainty about the effectiveness and effective duration of vaccine, and medical mistrust (24, 33, 35). Low income, ethnic minorities, young women, older people (75 years and above), political beliefs, and rural areas were some of the characteristics associated with hesitancy (30, 33, 36).

This study has several limitations. First, convenience and snowball sampling techniques were used to recruit the participants. The questionnaire was distributed *via* e-mail, social media, and WhatsApp which might have hampered its circulation among participants who might not be skilled in using these media or who may not prefer these media or apps leading to selection bias. However, this method of recruitment is pragmatic in the context of COVID-19 pandemic, considering the safety of participants and travel restrictions. Second, the study was not conducted among a cohort to have more robust estimation of change in trends. However, the participants were recruited from similar online spaces using similar sampling strategies, to minimize the potential differences between the participants in the three rounds of the surveys. The results of this study represent only the three points when the data were collected. The healthcare providers' willingness may have changed after the last phase of the study. Thirdly, the study failed to account to dissect the results based on the various categories of HCWs. It would be interesting if the data about the healthcare providers' specialty is also available. Despite these limitations, the repeated cross-sectional survey design was helpful in assessing the trends in willingness to receive COVID-19 vaccines over time. The observed substantial increase in acceptance during the survey periods needs to be further investigated to explore key barriers and facilitators of vaccine uptake.

This is one of the first studies in India to report the changes in willingness to accept COVID-19 vaccines among healthcare workers during the first and second waves of the SARS-CoV-2 pandemic. Willingness to receive COVID-19 vaccines among HCWs increased with time (between the first and third surveys), as the severity of the pandemic increased. To increase COVID-19 vaccine acceptance and coverage among HCWs, it is important to instill confidence in domestic vaccines and assist them to conduct accurate self-assessment of risk toward contracting COVID-19 infection. Perceived risk of infection, trust in the healthcare system and confidence in domestic vaccines were found to be significant predictors of uptake of COVID-19 vaccines. High vaccine acceptance among healthcare workers has the potential to improve acceptance in the general population as well. The study warrants multisectoral intervention for the improvement of vaccine acceptance among healthcare providers.

## Data availability statement

The raw data supporting the conclusions of this article will be made available by the authors, without undue reservation.

## Ethics statement

The studies involving human participants were reviewed and approved by Institutional Research Ethics Committee of Post-Graduate Institute of Medical Education and Research (PGIMER), Chandigarh, India. Written informed consent for participation was not required for this study in accordance with the national legislation and the institutional requirements.

## Author contributions

BPad, MG, and AKA conceptualized the study and designed the tools. BPad, LJ, JV, PS, VC, BPat, SK, RS, SP, SB, NR, VR, TK, KG, BM, LS, MG, and AKA conducted the study at national level and collected the data. All authors reviewed drafts, provided edits, and approved the final submission.

## Conflict of interest

The authors declare that the research was conducted in the absence of any commercial or financial relationships that could be construed as a potential conflict of interest.

## Publisher's note

All claims expressed in this article are solely those of the authors and do not necessarily represent those of their affiliated organizations, or those of the publisher, the editors and the reviewers. Any product that may be evaluated in this article, or claim that may be made by its manufacturer, is not guaranteed or endorsed by the publisher.

## Abbreviations

COVID-19: Coronavirus disease-2019
WHO: World Health organization.
